# Impact of Microbiota on Resistance to Ocular *Pseudomonas aeruginosa*-Induced Keratitis

**DOI:** 10.1371/journal.ppat.1005855

**Published:** 2016-09-22

**Authors:** Abirami Kugadas, Stig Hill Christiansen, Saiprasad Sankaranarayanan, Neeraj K. Surana, Stefanie Gauguet, Ryan Kunz, Raina Fichorova, Thomas Vorup-Jensen, Mihaela Gadjeva

**Affiliations:** 1 Department of Medicine, Division of Infectious Diseases, Brigham and Women’s Hospital, Harvard Medical School, Boston, Massachusetts, United States of America; 2 Department of Biomedicine, Aarhus University, Aarhus, Denmark; 3 Department of Medicine, Division of Infectious Diseases, Boston Children’s Hospital, Boston, Massachusetts, United States of America; 4 Department of Pediatrics and Anesthesia, UMass Memorial Children’s Medical Center, University of Massachusetts Medical School, Worcester, Massachusetts, United States of America; 5 Thermo Fisher Scientific Center for Multiplexed Proteomics, Harvard Medical School, Boston, Massachusetts, United States of America; 6 Laboratory of Genital Tract Biology, Department of Obstetrics, Gynecology and Reproductive Biology, Brigham and Women’s Hospital, Harvard Medical School, Boston, Massachusetts, United States of America; Wayne State University, UNITED STATES

## Abstract

The existence of the ocular microbiota has been reported but functional analyses to evaluate its significance in regulating ocular immunity are currently lacking. We compared the relative contribution of eye and gut commensals in regulating the ocular susceptibility to *Pseudomonas aeruginosa*–induced keratitis. We find that in health, the presence of microbiota strengthened the ocular innate immune barrier by significantly increasing the concentrations of immune effectors in the tear film, including secretory IgA and complement proteins. Consistent with this view, Swiss Webster (SW) mice that are typically resistant to *P*. *aeruginosa*–induced keratitis become susceptible due to the lack of microbiota. This was exemplified by increased corneal bacterial burden and elevated pathology of the germ free (GF) mice when compared to the conventionally maintained SW mice. The protective immunity was found to be dependent on both eye and gut microbiota with the eye microbiota having a moderate, but significant impact on the resistance to infection. These events were IL-1ß–dependent as corneal IL-1ß levels were decreased in the infected GF and antibiotic-treated mice when compared to the SPF controls, and neutralization of IL-1ß increased the ocular bacterial burden in the SPF mice. Monocolonizing GF mice with *Coagulase Negative Staphylococcus* sp. isolated from the conjunctival swabs was sufficient to restore resistance to infection. Cumulatively, these data underline a previously unappreciated role for microbiota in regulating susceptibility to ocular keratitis. We predict that these results will have significant implications for contact lens wearers, where alterations in the ocular commensal communities may render the ocular surface vulnerable to infections.

## Author Summary

Contact lens wear is associated with frequent *Pseudomonas aeruginosa*–induced keratitis, however the reasons for this association remain unclear. Recent genomics–based approaches revealed that contact lens wearers harbor altered ocular commensal communities when compared to non-lens wearers raising important questions, namely, does wearing of contact lenses increase the frequency of keratitis in patients due to contamination of the contact lenses with species derived from the skin or does ocular microbiota exert immune functions that are required for the maintenance of ocular health? We demonstrate a clear role for ocular microbiota in regulating protection against *Pseudomonas aeruginosa*–induced infections. At the ocular surface, commensal bacteria provide signals that regulate the magnitude of neutrophil recruitment during infection. These events may be driven by a frequent gram-positive commensal–*Coagulase Negative Staphylococcus (CNS)* sp. In addition to the impact of ocular microbiota, there is an important contribution of gut microbiota that stimulate neutrophil development in the bone marrow, thereby regulating the pool of mature neutrophils and their activation state. Cumulatively, these data show for the first time a role for microbiota in regulating the susceptibility to *P*. *aeruginosa*–keratitis.

## Introduction

The importance of microbiota in regulating lymphocytic development and inflammatory responses in the gut has been demonstrated in studies using germ-free (GF) or antibiotic-treated (ABX) mice [1–5]. Loss of intestinal microbiota diversity alters the host resistance to gut pathogens such as *Salmonella typhimurium*, *Listeria monocytogenes*, and *Clostridium difficile* to name a few [6–8]. Consistently, reconstitution of commensal bacterial communities facilitates the clearance of enteric opportunistic pathogens [9]. This suggests that transferring defined commensal bacterial populations into the host to re-establish microbiota offers an antibiotic–independent approach to combat infections. These approaches may not be exclusive for intestinal pathogens. A recent study demonstrated that antibiotic-treated mice showed increased sensitivity to viral infections. Housed under conventional conditions influenza virus–infected mice displayed lower viral titers and virus–associated mortality when compared to antibiotic–treated mice [10]. In lieu with these data, murine gut microbiota, particularly the *Segmented Filamentous bacteria*, promoted pulmonary type 17 immunity and resistance to *S*. *aureus* pneumonia [11]. Despite the growing understanding of the impact of the host–microbe alliance on immunity in the gastrointestinal tract, the extent to which individual microenvironments such as that of the eye are controlled by resident or distant microbiota remains unclear.

Unlike in the skin or gut, the ocular commensals are limited in abundance and richness. The most frequently identified species from the conjunctival surfaces in healthy humans are the *Coagulase Negative Staphylococci* sp. (*CNS* spp.), which include *Staphylococcus epidermidis* [12,13]. The less frequently present microbial species are *Propionibacterium* sp., *Corynebacterium* sp., *Staphylococcus aureus*, *Streptococcus* sp., *Micrococcus* sp., *Bacillus* sp., and *Lactobacillus* sp. [14]. While the Gram-positive species above are sparingly detected in ocular environment, the Gram-negative species are even less frequently detected and include *Pseudomonas aeruginosa*, *Enterobacter* sp., *Escherichia coli*, *Proteus* sp., and *Acinetobacter* sp. [12,15–19]. Numerically, the conjunctival surfaces harbor 10–100 CFU/swab in 20–80% of the swabs, a figure remarkably different from the number of commensals present in the oral mucosa where 100% of the swabs yield 10^7^−10^8^ CFU/ml of cultivatable bacterial species [20,21].

Utilizing 16S rRNA gene amplicon sequencing to analyze samples from contact lens wearers versus non–lens wearers, Shin *et al*. observed a shift of the conjunctival microbiota in the lens wearers towards relatively higher abundance of *Methylobacterium*, *Lactobacillus*, *Acinetobacter*, and *Pseudomonas*, and lower relative abundance of *Corynebacterium*, *Staphylococcus*, *Streptococcus*, and *Haemophilus*, suggesting that contact lens wearing alters the composition of ocular microbiota towards skin-like microbiota [22]. In agreement, the extended wear of contact lenses is associated with increased numbers of pathogenic organisms in conjunctival tissues [23–26]. Cumulatively, these studies raised important questions. Namely, how does ocular microbiota affect local immune responses to infectious pathogens; does wearing of contact lenses increase the frequency of keratitis in patients due to contamination of the contact lenses with species derived from the skin of the hand; and does ocular microbiota exert immune functions that are required for the maintenance of ocular health?

In the present study, we compared the impact of gut and ocular commensals on the susceptibility to *P*. *aeruginosa*-induced keratitis. We found that the GF Swiss Webster (SW) mice displayed a significantly higher susceptibility to *P*. *aeruginosa*–induced infection compared to conventional specific pathogen free (SPF) mice. This is indicated by higher bacterial burden and increased pathology scores in the corneas. Reducing the numbers of gut commensals significantly increased susceptibility to bacterial challenge, as did reduction of ocular commensals, albeit to a lesser effect. Mechanistically, the presence of microbiota elevated IL-1ß production in the *P*. *aeruginosa*-infected corneas. Monocolonizing GF mice with *CNS* sp. restored resistance to infection, implicating that *CNS* sp. are sufficient to induce protection against keratitis.

## Results

### Microbiota regulates ocular surface tear film protein composition

**Fig 1:**
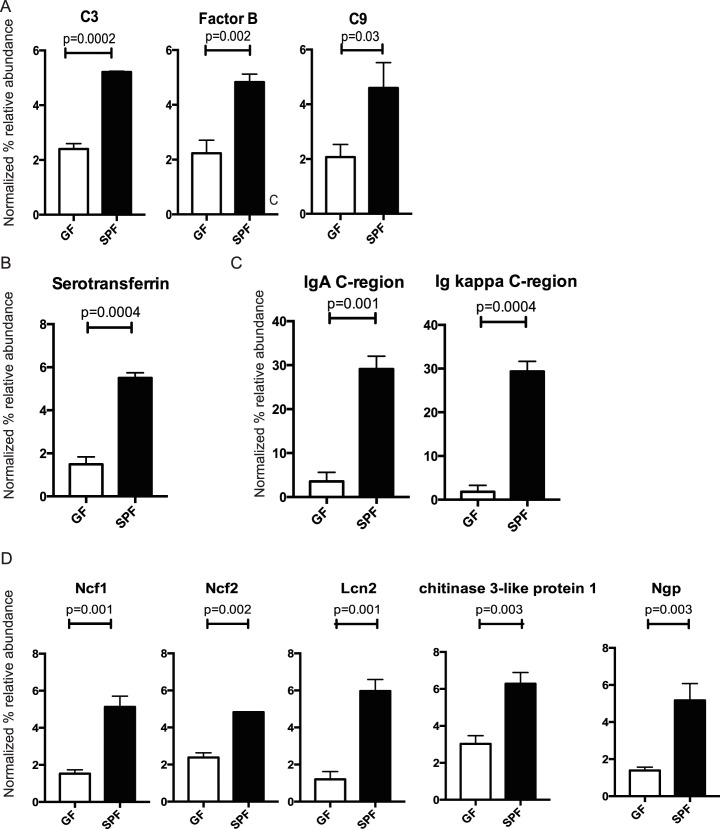
GF mice have decreased levels of innate and adaptive immune effectors at the ocular surface. A. Significantly decreased levels of complement proteins at the ocular surface in GF mice compared to SPF mice. B. Significantly decreased levels of iron binding protein serotransferrin at the ocular surface. C. Decreased levels of immunoglobulins at the ocular surface. D. Decreased levels of neutrophil-derived peptides at the ocular surface. Significant differences were detected in the levels of nuclear cytosolic protein 1 (Ncf1) and 2 (Ncf 2), myeloid bactenecin (ngp), neutrophil galatinase associated lipocalin (Lcn2), and chitinase-3-like protein 1. Ocular surface washes were pooled from GF (n = 5) and SPF mice (n = 5). Two-three biological replicates were analyzed per experiment. Five μg total protein were digested with trypsin, peptides were labeled, multiplexed with TMT, and quantified using LC-MS^3^. p-values were calculated using Benjamini-Hochberg FDR correction.

To evaluate how microbiota alters baseline ocular immune barrier, eye washes were collected from GF and SPF SW mice and their protein signature was characterized using quantitative LC-MS/MS (S1 Table). The total protein levels of the ocular washes obtained from GF and SPF SW mice were comparable (S2 Fig). Peptides derived from 63 proteins were differentially present in SPF SW mice compared to GF SW mice at baseline (S2 Table). Important innate immune effectors including complement component C3, factor B, and complement component C9 were significantly reduced in GF-derived tear films (Fig 1A, S2 Table). Iron-scavenging protein like serotransferrin was also significantly reduced, suggesting decreased baseline anti-microbial activity (Fig 1B and S2 Table). Expectedly, peptides derived from Ig kappa constant region and IgA heavy chain- constant region were significantly reduced in GF mice (Fig 1C). Interestingly, neutrophil-derived peptides such as lipocalin 1 (Lcn2), neutrophil cytosolic factor (Ncf2), neutrophil cytosolic factor (Ncf1), chitinase-3-like protein 1 (Fig 1D, for complete list see S2 Table) were less abundant in GF-derived tear films compared to SPF-derived washes, illustrating decreased neutrophil trafficking to the ocular surfaces at steady state in the absence of microbiota. In contrast, only 8 identified proteins were upregulated in GF-derived ocular washes when compared to SPF (S3 Table), neither of which had previously identified antimicrobial activity. Cumulatively, these data demonstrated compromised innate and adaptive effectors at the ocular surface in the absence of microbiota and suggested increased susceptibility to infection.

### GF mice are more susceptible to *Pseudomonas aeruginosa* eye infection than SPF mice

**Fig 2:**
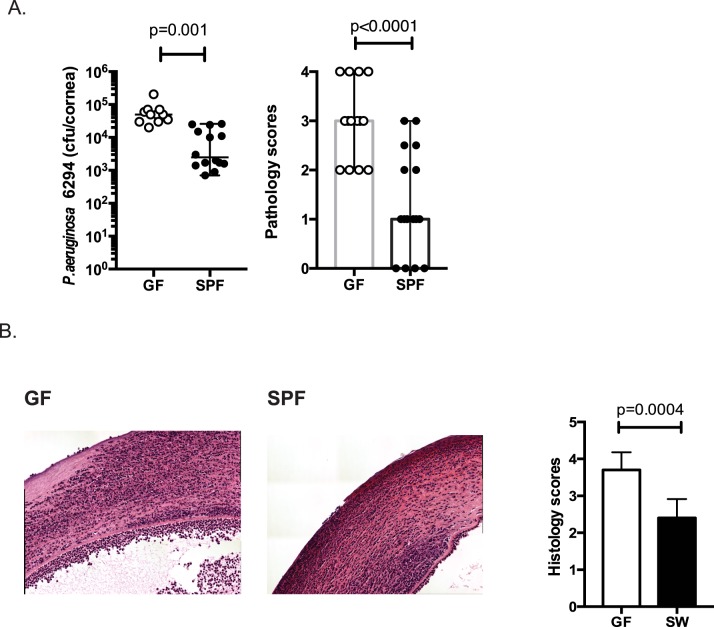
GF mice are more susceptible to ocular *P*. *aeruginosa*–induced keratitis compared to SPF mice. A. *P*. *aeruginosa* 6294 CFU/cornea (left) and pathology scores (right) 24 h following infection. Groups of GF (n = 11) and SPF SW (n = 14) mice were infected with 1×10^7^ CFU *P*. *aeruginosa* per eye. Data were pooled from two independent experiments performed under comparable conditions. p-values were generated using Student’s *t*-test. B. Hematoxylin-eosin staining of sections derived from GF and SPF SW mice. Images are taken at 10x magnification to illustrate pathological alterations during infection. Data are representative images taken from the infected eyes of GF mice (n = 4) and SPF (n = 4) mice. The quantification of histopathological changes confirmed increased pathology in GF corneas when compared to SPF. p-values by Mann-Whitney test.

**Fig 3:**
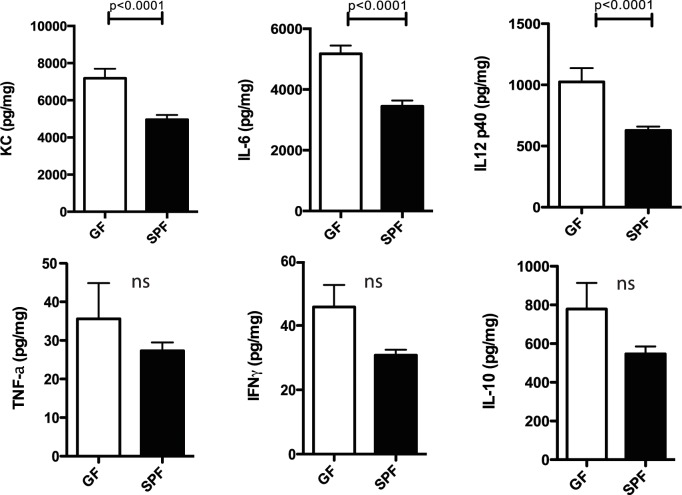
Inflammatory responses in the corneas of GF SW mice are increased when compared to SPF mice infected with *P*. *aeruginosa* strain 6294. Groups of 14 GF mice and 14 SPF mice were infected with 1×10^7^ CFU *P*. *aeruginosa* placed onto scratch-injured eyes. Corneas were harvested at 24 h after infection, washed in F12 media, homogenized in PBS containing a mix of protease inhibitors and supplemented with 0.5% Triton to disrupt plasma membranes. The levels of cytokines in corneal lysates were measured using a Meso Scale Discovery (MSD) multiplex assay and compared with Bonferroni’s correction for multiple comparisons (p<0.008). IL-12p40, IL-6 and KC were significantly increased in GF mice when compared to SPF mice p = 0.0001, p = 0.0001, and p = 0.0001, respectively. Data pooled from two experiments performed under comparable conditions.

To determine the effect of the presence of microbiota on *P*. *aeruginosa*–induced keratitis, GF and SPF SW mice were infected with *P*. *aeruginosa* strain 6294. At 24 h after infection corneas of infected GF SW mice exhibited a significantly higher bacterial burden (p = 0.01, Student’s *t*-test) than conventionally maintained control animals (Fig 2A). This correlated with increased histopathology scores in the GF mice (Fig 2B, p = 0.0004, Mann-Whitney test). Comparison of the inflammatory profiles of infected GF and SPF SW mice is shown in Fig 3. A panel of cytokines, TNF-α, KC, IL-6, IL-12p40, IFN-γ, and IL-10 were quantified at the protein level in infected corneas harvested from the individual animals. Corneal homogenates of infected GF mice contained significantly higher levels of IL-6 (p = 0.0001), IL-12 p40 (p<0.0001), and KC (p = 0.0001) compared to the infected SPF mice corneas. There were no significant differences in the levels of TNF-α, IFN-γ, or the anti-inflammatory cytokine IL-10, although there was a tendency for increased presence in the GF mice (Fig 3). These data showed elevated proinflammatory responses to infection in the absence of microbiota.

**Fig 4:**
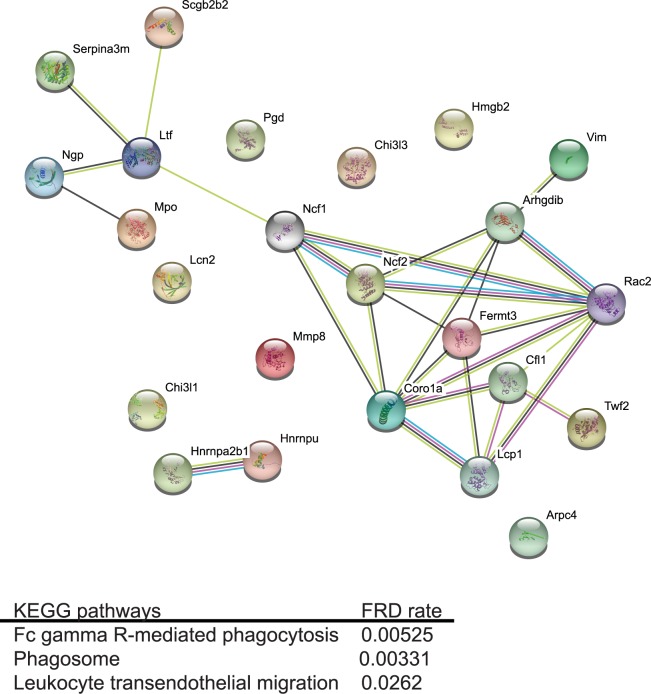
Neutrophil recruitment to the corneas of infected GF SW mice is increased at the peak of infection. Ocular surface washes were collected from *P*. *aeruginosa*-infected GF (n = 5) and SPF SW (n = 5) mice. 5 μg total protein was in-gel digested, trypsinized, labeled, and profiled using LC-MS/MS. Significantly upregulated proteins in the infected GF mice were visualized using String-based web software and revealed enrichment for proteins associated with phagocytosis (FDR 0.000525), phagosome (FDR 0.003), leukocyte trafficking (FDR 0.02) and high degree of protein-protein interaction (PPI enrichment p value 7.55e-15).

Further, quantitative proteomic analysis of the tear film from the infected GF and SPF mice showed that *P*. *aeruginosa* infection induced differential upregulation of 293 proteins in the GF group compared to the non-infected GF baseline, whereas only 106 proteins were upregulated in the SPF group when compared to the non-infected baseline. When comparing the protein signature of infected GF mice to that of infected SPF mice, 24 proteins were differentially present including proteins with antimicrobial activity (e.g., lactotransferrin, myeloid bactenecin, neutrophil gelatinase-associated lipocalin), and proteins with enzymatic activity (e.g., neutrophil collagenase and myeloperoxidase) (S4 Table). String-based analysis of the upregulated proteins showed enrichment for phagocytosis, phagosome, and leukocyte transmigration KEGG pathways (Fig 4), further supporting the observation of elevated susceptibility of GF mice to infection. Cumulatively, these data demonstrate that GF SW mice are capable of mounting a protective anti-*P*. *aeruginosa* immune response by stimulating increased recruitment of neutrophils to the site of infection despite the initially low innate immune response.

### Local microbiota affects the resistance to *P*. *aeruginosa*–induced ocular infection

**Fig 5:**
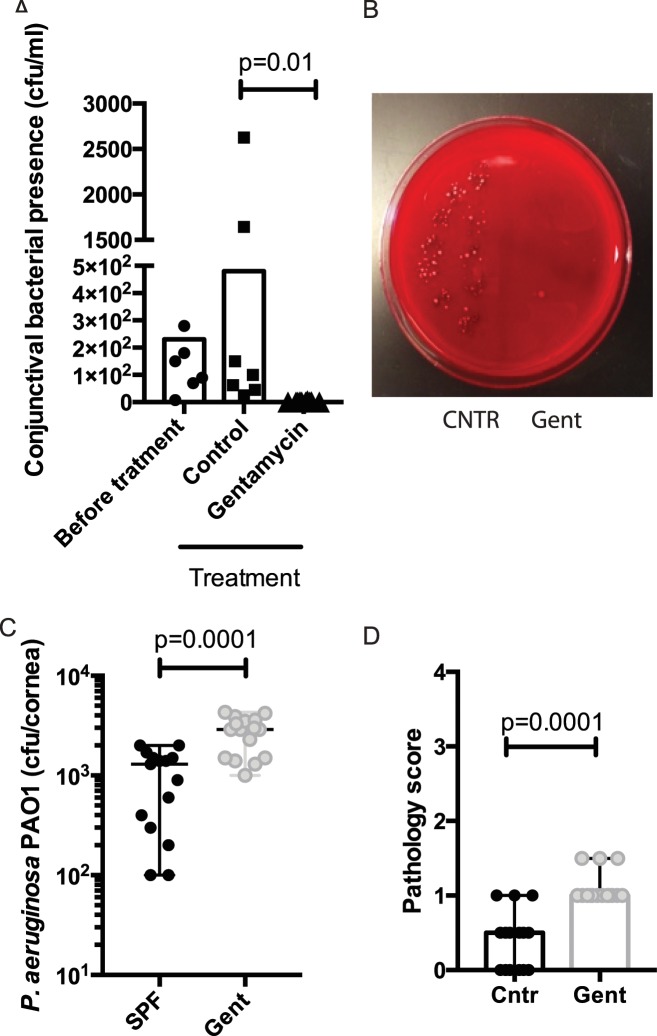
Topical gentamycin treatment reduces the resistance to *P*. *aeruginosa*-induced keratitis. **A.** Commensal conjunctival presence in SPF mice (n = 10) is significantly higher than in mice (n = 10) treated topically with gentamycin ointment for 3 days prior to the infectious challenge. Data were pooled from two independent experiments. Groups were compared with one-way ANOVA, p = 0.01. **B.** Detection of bacterial growth from conjunctival swabs on a blood agar plate. The left side of the plate was seeded with 10 μl/spot suspension derived from the conjunctival swabs of control mice, the right side of the plate was seeded with 10 μl/spot suspension derived from the conjunctival swabs of Gentamycin-treated mice. **C.** Corneal bacterial burden after challenge with *P*. *aeruginosa PAO1* is moderately but significantly increased in the Gentamycin–treated cohort. Data were pooled from three performed experiments. Unpaired Student’s *t*-test. **D.** Pathology scores. p-values are derived by Mann-Whitney test.

To evaluate the impact of ocular microbiota on regulating the resistance to keratitis, separate cohorts of SPF SW mice were pretreated topically with gentamycin to reduce the numbers of commensals prior to infection. The recoverable cultivatable conjunctival bacterial presence included from SPF SW mice included mannitol-fermenting and non-fermenting *Staphylococcus* spp. and minor proportion of other species such as *Streptococcus* sp. (Fig 5). The presence of these bacterial commensals was completely ablated after a 4-day topical treatment with gentamycin (Fig 5). Upon infection, the corneas of gentamycin-treated mice displayed moderate, but significantly higher numbers of *P*. *aeruginosa* (p = 0.0001, Student’s *t*-test) compared to age- and gender-matched control mice. Correspondingly, the pathology scores were significantly increased in the topical treatment group when compared to the controls (p = 0.0001, Mann-Whitney test), illustrating that the ocular commensals contributed to the resistance to infection.

### Oral antibiotic treatment increases susceptibility to *P*. *aeruginosa*–induced ocular infection

**Fig 6:**
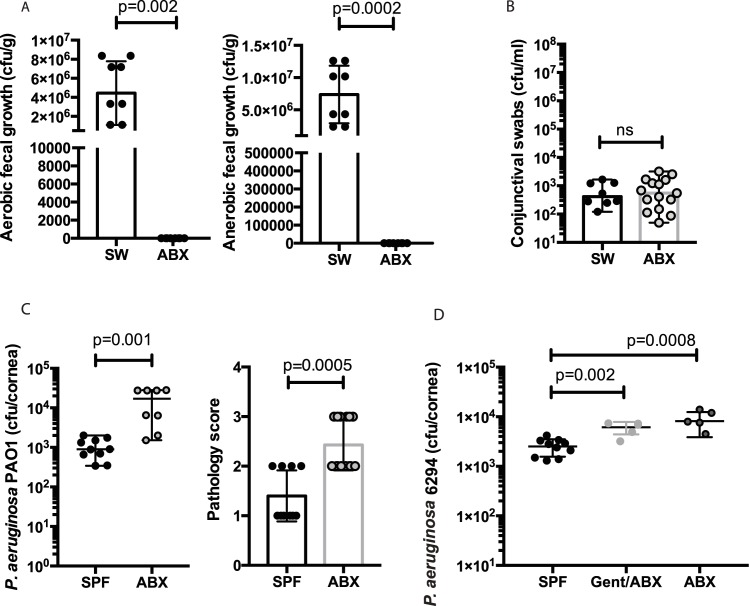
Oral antibiotic treatment significantly reduces the resistance to *P*. *aeruginosa*-induced infection. **A.** Aerobic and anaerobic gut commensal bacterial burden in SPF mice after oral antibiotic treatment shows significant decrease in gut commensal presence. Circles indicate individual animals. p-values by unpaired Student’s *t*-test. **B.** Commensal conjunctival presence is not affected in mice treated with oral antibiotics. Circles indicate individual animals. **C.** Pathology scores and corneal bacterial burden at 24h after infection. Groups of 10 SPF SW mice and 10 ABX mice were infected with 1×10^7^ CFU *P*. *aeruginosa* PAO1. Mouse corneas were harvested at 24 h after infection. ABX mice had significantly higher pathology scores and bacterial burdens indicating reduced resistance to infection. p- values by Student’s *t*-test (CFU) and Mann-Whitney test (pathology). Data were pooled from two experiments performed. **D.** Corneal bacterial *P*. *aeruginosa* 6294 burden measured at 24h after the infectious challenge in cohorts of SPF mice, mice treated locally with gentamycin and orally with antibiotics (gent/ABX), and mice treated orally with antibiotics. ABX mice treated with gentamycin showed comparable bacterial levels to the ABX mice that received no additional topical antibiotic treatment. Data were pooled from two experiments performed. The circles represent individual mice. p-values by one-way ANOVA (p<0.0001) followed by Turkey’s multiple comparisons test.

Next, we evaluated the requirement for non-ocular microbiota in regulating the susceptibility to bacterial keratitis. SPF SW mice were treated orally with an antibiotic cocktail before the infections experiments, which resulted in a significant decrease in numbers of gut microbiota (Fig 6A) while preserving the ocular commensals in the conjunctiva (Fig 6B). ABX mice showed increased susceptibility to ocular *P*. *aeruginosa* challenge, which was exemplified by elevated bacterial burden in the corneas (p = 0.001, Student’s *t*-test) and increased corneal pathology (p = 0.0005, Mann-Whitney test) (Fig 6C). Combined treatment of SPF mice with gentamycin and oral antibiotics did not result in increased susceptibility to infection when compared to infected mice treated with oral antibiotics alone, demonstrating a non-cumulative effect (Fig 6D).

**Fig 7:**
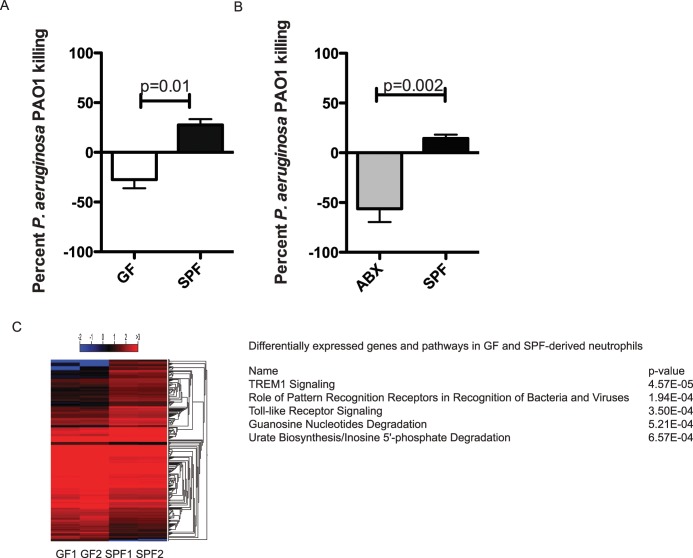
PMNs derived from either GF mice or mice treated with oral antibiotics have significantly decreased bactericidal activities against *P*. *aeruginosa* and altered transcriptomic signatures. **A.** Percent *P*. *aeruginosa* PAO1 killing by GF PMNs and SPF SW-derived PMNs *in vitro* (p = 0.01, Unpaired Student *t*-test). Bone marrow-derived mature GF and SPF SW PMNs were exposed to *P*. *aeruginosa* PAO1 for 90 min at 37°C. Upon completion of incubation, an aliquot from the reaction mixture was plated to quantify the remaining PAO1. Neutrophils derived from animals without commensal microbiota display significantly reduced bactericidal capacity against *P*. *aeruginosa*. **B.** Percent of *P*. *aeruginosa* PAO1 killing by SPF SW-derived PMNs and PMNs purified from mice treated with oral antibiotic cocktail *in vitro* (p = 0.002, unpaired Student *t*-test). Neutrophils derived from mice that received oral antibiotics display significantly reduced bactericidal capacity against *P*. *aeruginosa*, illustrating that microbiota-derived signals promote neutrophil function. **C.** GF-derived neutrophils have significantly different transcriptional signature when compared to SPF-derived neutrophils. Shown are the transcriptomic profiles of differentially expressed genes in GF- and SPF-derived PMNs obtained by RNAseq analysis and the predicted pathways involved in PMN maturation in a heat map of the differentially expressed genes in GF- and SPF-derived PMNs. Hierarchical cluster analysis was performed on CLC Genomics workbench on subset of transcripts that show differential presence between the GF- and SPF-derived neutrophils (≥ 1.5-fold; p<0.05). The same subset was used to predict the potential upstream regulators by IPA to identify pathways that are involved in priming neutrophils for optimal function.

Consistent with these observations, baseline bactericidal properties of neutrophils derived either from GF and ABX mice were significantly reduced compared to SPF mice (Fig 7). GF-derived BM PMNs exhibited 50% reduced killing of *P*. *aeruginosa PAO1* (Fig 7A) and *PA14* (S3 Fig) when compared to PMNs isolated from the SPF SW animals. Reactive oxygen species (ROS) released by GF PMNs in response to *P*. *aeruginosa* 6294 were significantly less than those released by the SPF PMNs (S4 Fig).

To further characterize the phenotype of GF-derived neutrophils, RNA sequencing experiments were carried out using bone marrow derived purified mature Ly6G^+^, CD11b^+^ PMNs (Fig 7C). Similar total number of reads was obtained from the individual biological duplicates. Upon mapping of these reads to the reference genome database, approximately 270 differentially expressed transcripts were identified (S5 Table). Ingenuity pathway–based prediction for upstream regulators suggested the involvement of the LPS (p = 5.95E-19), IFN type 1 (p = 3.36E-18), IL-1ß (8.93E-15), ACKR (1.16E-14), TNF-α (2.57E-14), MyD88 (p = 2.7E-13), TICAM (p = 9.6E-13), and IFN-γ (p = 6.24E-12) pathways. These data demonstrated significant phenotypic alterations between the GF- and SPF-derived neutrophils and support the conclusion that microbiota promotes neutrophil bactericidal activities against *P*. *aeruginosa*.

### Microbiota-driven IL-1Ββ responses promote resistance to *P*. *aeruginosa*–induced keratitis

**Fig 8:**
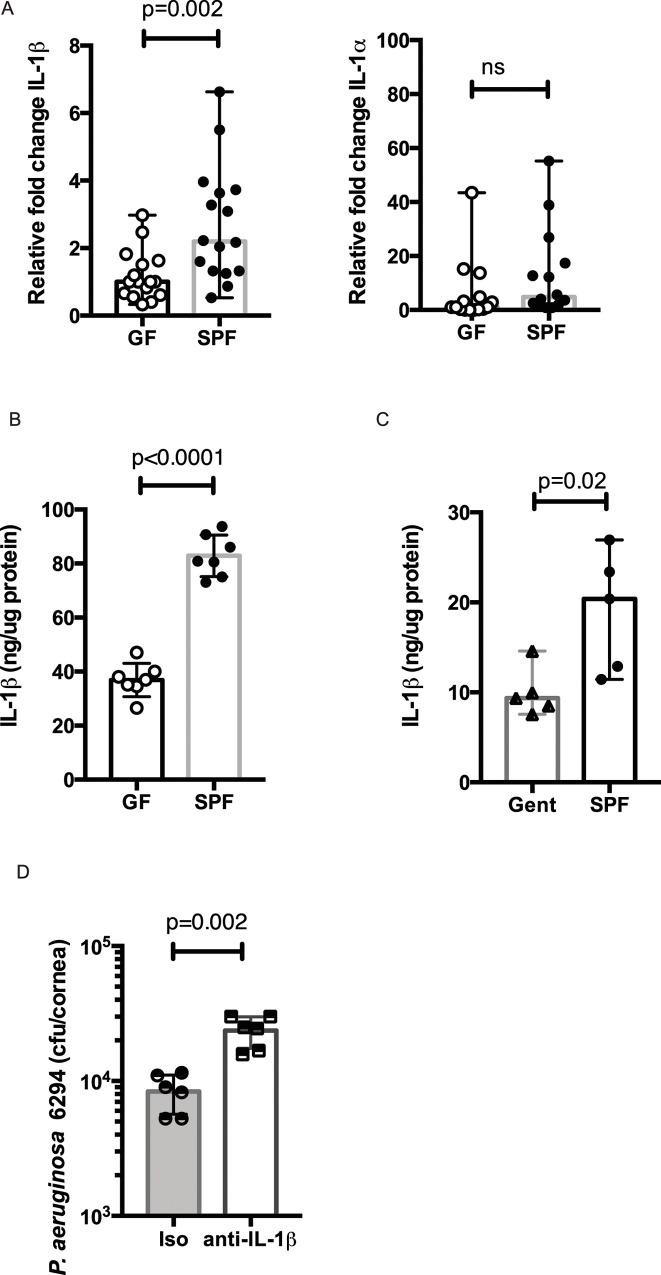
Commensal presence promotes IL-1ß release during infection. **A.** Differential presence of IL-1ß transcripts in the conjunctival tissues of GF and SPF SW mice. Quantitative RT-PCR for IL-1ß and IL-1α transcripts were carried out using conjunctival tissues from GF and SPF mice. GF conjunctival tissues have significantly lower levels of IL-1ß transcripts when compared to SPF-derived tissues. Data were pooled from two independent experiments and plotted as median values with range. The circles represent individual mice. p-values by Student’s *t*-test. Bonferroni correction for multiple comparisons p = 0.025.**B**. IL-1β protein levels in the corneal tissues of infected GF and SPF mice with *P*. *aeruginosa* 6294. Groups of GF (n = 5) and SPF-SW (n = 5) mice were infected with 1×10^7^ CFU *P*. *aeruginosa* 6294 onto eyes. Infected GF mice mount significantly less IL-1ß when compared to SPF mice. Data were pooled from two experiments. p-values by Student’s *t*-test. **C.** IL-1ß levels measured by ELISA in the corneal tissues of either mice treated topically with gentamycin or control mice infected with *P*. *aeruginosa PA01*. Topical gentamycin treatment significantly reduced the levels of IL-1ß released during infection. Data were median values with range. p-values by Student’s *t*-test. **D.** Neutralization of IL-1ß increased corneal bacterial burden in the resistant SPF SW mice (n = 6) when compared to the control mice (n = 6). p-values by Student’s *t*-test. Data are pooled from two performed experiments.

Since deficiency in the IL-1ß signaling pathways sensitizes to *P*. *aeruginosa*–induced infections [27], ocular IL-1ß levels were quantified at baseline and during *P*. *aeruginosa*-induced infection. The levels of the IL-1ß mRNA transcripts in the GF-derived conjunctival tissues were two-fold less than in the SPF-derived tissues (p = 0.002, Student’s *t*-test). This was in contrast to the levels of IL-1α RNA transcripts, which were comparable in both groups of mice (Fig 8A). Consistent with differential microbiota-driven priming, GF mice mounted a significantly weaker IL-1ß response when compared to the SPF mice (Fig 8B, p = 0.0001, Student’s *t*-test) during infection with *P*. *aeruginosa 6294*. In contrast to the levels of IL-1ß, other proinflammatory cytokines, which are typically upregulated during bacterial keratitis, like IL-6, KC, and IL-12p40 levels were significantly increased in infected corneal tissues from GF mice (Fig 3). Importantly, local gentamycin treatment resulted in two-fold lower IL-1ß levels in *P*. *aeruginosa* PAO1-infected tissues (Fig 8C, p = 0.02, Student’s *t*-test), illustrating that local microbiota promoted the magnitude of IL-1ß released during infection at the ocular mucosa. Lastly, neutralizing anti-IL-1ß antibody in the naturally more resistant SPF SW mice prior to infection led to an increase in corneal *P*. *aeruginosa* burden (Fig 8D, p = 0.002, Student’s *t*-test) confirming the requirement for IL-1ß in regulating susceptibility to infection.

### Monocolonizing GF mice with *Coagulase Negative Staphylococcus* sp. is sufficient to restore resistance to ocular infection

**Fig 9:**
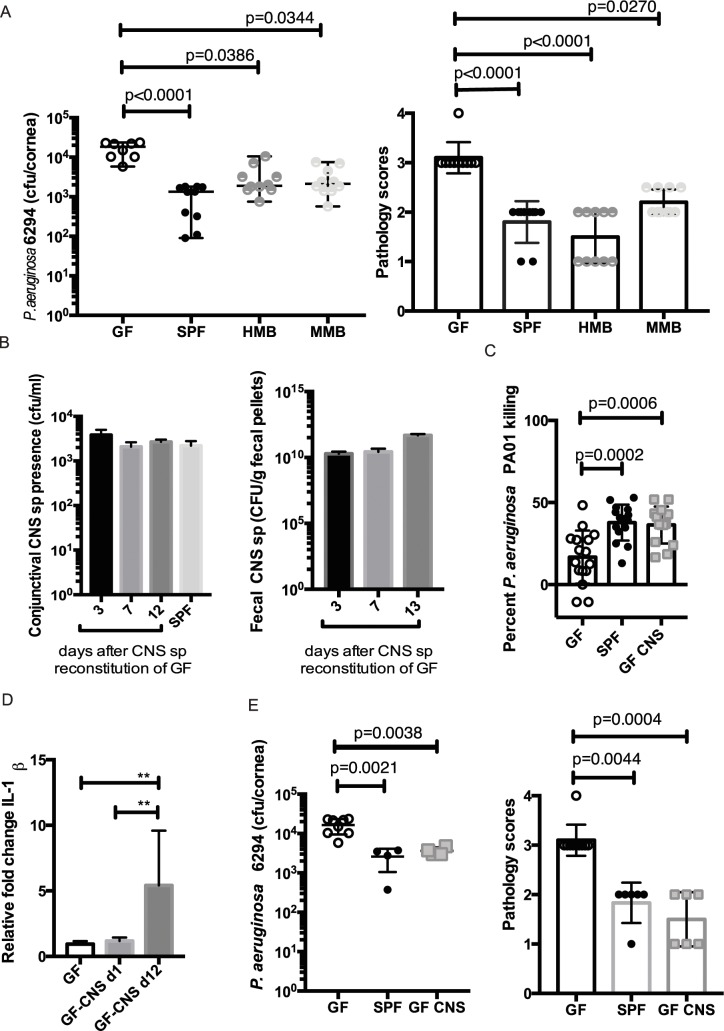
*CNS sp* restore resistance to ocular *P*. *aerugiunosa* 6294–induced keratitis. **A.** *P*. *aeruginosa* 6294 CFU/cornea (left) and pathology scores (right) 24 h following infection. Groups of GF (n = 8), SPF-SW (n = 10), HMB (n = 10), and MMB (n = 10) mice were infected with 10^7^ CFU per eye. Data were pooled from two performed experiments. The bacterial burden data and pathology scores were analyzed with one-way ANOVA (p<0.0001) followed by Kruskal-Wallis multiple comparisons test. The individual circles represent individual animals. Data suggest that reconstituting GF mice with either human-derived or mouse-derived gut commensals restores resistance to infection. **B.** Monocolonizing GF mice with *CNS* sp. by topical application of *CNS* sp. suspension onto the eye restores resistance to infection. Quantification of the levels of *CNS* sp recovered from the conjunctival swabs one week and 12 days post-reconstitution. Quantification of the *CNS* sp. in the fecal pellets, demonstrating stable monoassociation. **C.** Neutrophils derived from monocolonized GF mice show comparable bactericidal activity against *P*. *aeruginosa PAO1* as SPF-derived PMNs. p-values by one-way ANOVA (p<0.0001) followed by Kruskal-Wallis multiple comparisons test. The individual sample values are plotted as circles. **D**. Conjunctival *CNS* sp. presence promotes IL-1ß transcription. Conjunctival tissues from GF (n = 5) and monocolonized GF mice with *CNS* sp. (n = 3) were collected, total RNA purified, and the levels of IL-1ß transcripts quantified with RT-PCR. The levels of transcripts were compared using one-way ANOVA followed by Turkey’s multiple comparisons test. Significant p<0.05 values are indicated by asterisks. The tissues harvested from the monocolonized mice showed significantly increased presence of IL-1ß transcripts 12 days post-colonization. **E.** *P*. *aeruginosa* 6294 CFU/cornea (left) and pathology scores (right) 24 h following infection in monocolonized GF with *CNS* sp. (n = 6), SPF SW (n = 5), or GF (n = 10) mice. Monocolonizing of GF mice with *CNS* sp. restores the resistance to infection. The values for the individual animals are represented as circles. Significant differences are denoted with asterisks. p-values by one-way ANOVA followed (p = 0.0007) by ordinary one-way ANOVA multiple comparisons test. Pathology scores were compared using one-way ANOVA (p<0.0001) followed by Kruskal-Wallis multiple comparison test.

To evaluate which microbial communities or individual commensal species promote ocular health, GF mice were reconstituted with either mouse- or human-derived gut microbiota. The reconstituted mice showed less susceptibility to *P*. *aeruginosa* 6294, reduced pathology, and decreased bacterial presence in the cornea (Fig 9A). While the gut microbiota in these reconstitution experiments was of different origin: mouse- or human–derived, all reconstituted animals retained *CNS* sp. at the ocular surface. To evaluate the impact of the *CNS* sp. on regulating susceptibility to keratitis, monocolonization experiments were carried out.

A single topical application of *CNS* sp. was sufficient to colonize the ocular surface. *CNS* sp. were recovered two weeks after the initial commensal exposure from the cornea as well as from feces of monocolonized mice, indicating gut colonization in these animals (Fig 9B). The monocolonized mice showed restored PMN bactericidal properties against *P*. *aeruginosa* (Fig 9C), increased IL-1ß conjunctival transcripts (Fig 9D) and similar resistance to *P*. *aeruginosa*-induced infection as the SW mice (Fig 9E). Cumulatively, these data underline the importance of *CNS* sp. in regulating neutrophil activation and resistance to *P*. *aeruginosa*-induced keratitis.

## Discussion

Unlike any other body site, the ocular mucosal surfaces harbor very few cultivatable bacterial species [17,21]. A lower percentage of the conjunctival swabs give rise to cultivatable bacteria that are in stark contrast to the number of recovered bacteria from the skin or oral mucosa where 100% of the swabs result in microbial growth [28–30]. The cultivatable commensal species from the eye are limited in repertoire and include *Coagulase Negative Staphylococcus* sp., *Propionibacterium* sp., *Corynebacterium* sp., *S*. *aureus*, *Streptococcus* spp., *Micrococcus* sp., *Bacillus* sp., and *Lactobacillus* sp. [12,15–19]. These observations prompted the inquiry into whether small numbers of bacterial species have measurable and significant impact on ocular immunity [21]. We examined this question by comparing the ocular immune responses to *P*. *aeruginosa*, a frequent opportunistic ocular pathogen, in GF mice, in mice treated with topically applied antibiotics to reduce the ocular microbiota, in mice treated with oral antibiotics to reduce gut microbiota, and in GF mice monocolonized with *CNS* sp.

First, we examined whether depletion or absence of microbiota leads to increased susceptibility to *P*. *aeruginosa*–induced keratitis in naturally resistant SW mice and, thereby, to altering the degree and quality of the commensal-driven immune priming. We found that GF mice were significantly more susceptible to ocular challenge with *P*. *aeruginosa* compared to SPF-maintained SW mice. This was exemplified by increased bacterial presence in the cornea, elevated inflammatory cytokine responses, and higher ocular pathology scores at the peak of infection (Fig 2). These data reveal the importance of microbiota in regulating corneal resistance to *P*. *aeruginosa*.

Local treatment with gentamycin, and thereby reduction of local microbiota, elevated the susceptibility to infection in SPF SW mice demonstrating that local microbiota plays a measurable and significant, albeit moderate, role in maintaining ocular immunity (Fig 5). There are significant implications that stem from these findings. In the USA, one in 2,500 daily contact lens wearers develops *P*. *aeruginosa* keratitis. Therefore, there is a long-lasting interest in the understanding of how contact lenses predispose to this infection. Recently, Shin *et al*. reported that contact lens wear significantly alters the conjunctival commensal community [22]. Interestingly, the alterations were associated with reduced abundance of *Corynebacterium* sp., *Staphylococcus* sp., *Streptococcus* sp., and *Haemophilus* sp. in the contact lens wearers [22]. However, the biological implications of this difference are uncertain. Based on our results, we propose that contact lenses are not simply a vector for pathogenic organisms but that their use lowers immune effector responses elicited by commensal microbiota, and that this sensitizes to infection. We hope that our data will lead to exploring novel designs and regimens of contact lens wear to achieve minimal impact on the commensal communities, thereby decreasing complications by infections.

Previous studies have demonstrated that deficiency in IL-1ß signaling rendered the C57BL/6 mice more susceptible to *P*. *aeruginosa*–induced eye infection [31]. In these experiments, IL-1ß was released by corneal macrophages in response to *P*. *aeruginosa*–stimulated TLR4 and TLR5 activation and by neutrophils [27,31]. Consequently, IL-1ß –dependent signaling promoted neutrophil recruitment during keratitis and *P*. *aeruginosa*–induced pneumonias [31–35]. Our data not only confirm the critical role of IL-1ß signaling in mediating protection against *P*. *aeruginosa*–induced keratitis by converting the resistant SW mice to susceptible (Fig 8D) but also show that microbiota regulates the magnitude of IL-1ß released during infection. Consistently, GF mice displayed fewer IL-1ß transcripts (Fig 8A) and, conversely, monocolonizing GF mice with *CNS* sp. increased IL-1ß transcript levels (Fig 8D). Currently, the type of the cells that produce IL-1ß transcripts is under investigation and includes epithelial cells, conjunctival antigen presenting cells, and goblet cells as they are exposed to commensal-derived products.

The ability of commensals to prompt IL-1ß –driven responses has been previously recognized at mucosal sites exposed to much more prominent commensal presence such as the gut [36,37]. Vancomycin-sensitive Gram-positive commensals promoted the expansion of IL-1R1+ γδ T cells, which elicited protection against peritoneal *E*. *coli* infection via improved neutrophil recruitment. In the skin, *S*. *epidermidis* primed the CD11b^+^ CD11c^+^ dendritic cell–derived IL-1ß production, thereby promoting the maintenance of CD8^+^ and CD4^+^ T cells [37]. Exposure of *S*. *epidermidis*–loaded dendritic cells to CD8^+^T cells triggered potent IL-17A and IFN-γ production, which had protective consequences against *Candida albicans* challenge. Whether microbiota-driven γδT cells or adaptive CD8^+^, CD4^+^ T cells are critical in regulating PMN recruitment during infection in the ocular mucosa remains to be clarified.

While depletion of the local microbiota resulted in a moderate increase in the susceptibility to infection, the reduction of gut microbiota had a stronger impact. Of note, when oral antibiotics were combined with topical gentamycin treatment to ablate the conjunctival commensal presence, no significant differences were observed in the recovered corneal *P*. *aeruginosa* burden between the different treatment groups, suggesting that the pathways were not synergistic (Fig 6D).

The observation that the ocular microbiota was less efficacious in regulating resistance to infection when compared to gut microbiota, depends on gut microbiota–driven neutrophil maturation. Depletion of gut microbiota significantly reduced neutrophil maturation and bactericidal activities against *P*. *aeruginosa* (Fig 7). These data are consistent with recent publications reporting that GF mice had reduced proportions and differentiation potential of the granulocytic progenitors in the bone marrow which consequently rendered them susceptible to *Listeria monocytogenes*, *Streptococcus pneumoniae*, *Staphylococcus aureus*, *and Escherichia coli* infections [38–41]. The observation that the reconstitution with mouse or human gut derived microbiota or monocolonizing the animals with *CNS* sp. restored the bactericidal properties of PMNs against *P*. *aeruginosa* (Fig 9) is also in accord with the recently published results by Balmer *et al*. where reconstitution experiments with *E*. *faecalis*, *E*. *coli K-12*, and *S*. *xylosus* were sufficient to restore neutrophil maturation [38]. Aiming at identifying microbiota-derived molecules and, ultimately, the underlined pathways, serum transfer experiments from SPF mice to GF mice pointed to bacteria-derived, heat stable, soluble compounds that triggered TLR signaling [38]. “Triple” colonization of MyD88^-/-^/TICAM^-/-^ mice with *E*. *faecalis*, *E*. *coli K-12*, and *S*. *xylosus* failed to stimulate granulopoiesis highlighting the importance of commensal-induced MyD88 signaling. Intriguingly, the transcriptomic signature of GF-derived neutrophils suggest that these neutrophils can respond to LPS, IL-1ß, and type I and type II interferon challenges, thereby providing evidence that neutrophils display significant degree of transcriptional plasticity when generated in the bone marrow. Consistently, challenges of GF mice with either *E*. *coli*-derived LPS or peptidoglycan enhanced the neutrophil priming [40,41]. Cumulatively, these data suggest that tonic gut microbiota–derived signals not only stimulate neutrophil survival as documented in [39], but also increase the bactericidal activity of neutrophils.

Neutrophil trafficking to the ocular surfaces at steady state has been reported and is considered to promote resistance to infections, however the stimuli that trigger neutrophil recruitment are not known [42–44]. The LC-MS^3^ data clearly show increased neutrophil presence at the ocular surfaces of conventionally housed SPF mice, which was less pronounced in GF mice (Fig 1). Consistently, there was a delayed initial recruitment of PMNs to the eye in the GF mice during *P*. *aeruginosa*–induced infection, which as disease progressed resulted in a more significant pathology at the later time points (Fig 2). These data are congruent with previous results showing delayed neutrophil recruitment to the cornea in the *Staphylococcus aureus* infected GF mice [45], and further elucidate the importance of microbiota-driven neutrophil recruitment to the ocular surfaces.

In conclusion, we demonstrate a clear role for microbiota in regulating the immune response at the ocular surface. Mechanistically, gut microbiota primes the development and activation of neutrophils in the bone marrow, thereby regulating the pool of mature neutrophils and their activation state. Locally, microbiota provides signals that regulate the magnitude of neutrophil recruitment to the ocular tissues during infection in an IL-1ß –dependent manner. Identifying the commensal species and the biochemical composition of microbiota-induced signals that regulate neutrophil recruitment and bactericidal activities will allow development of new antibiotic-adjunctive therapeutic approaches.

## Materials and Methods

### Ethics statement

All animal experiments were performed following National Institutes of Health guidelines for housing and care of laboratory animals and performed in accordance with institutional regulations after protocol review and approval by the Harvard Medical School Animal Care and Use Committee and were consistent with the Association for Research in Vision and Ophthalmology guidelines for studies in animals (protocol 404R98).

### Mice

Mice were housed and bred in the Channing Laboratory Animal Care Facilities. The SW GF mice were purchased from the Gnotobiotic Core Facility, Harvard Medical School. Age and gender matched SW mice were purchased from Taconic Farms. 8–10 week old, gender-matched mice were used throughout the experiments.

### Bacterial strains and inocula

Invasive *P*. *aeruginosa* strains 6294 and PAO1 were used throughout these experiments. The bacterial strains were grown overnight at 37°C on Tryptic Soy Broth agar plates supplemented with 5% sheep blood. The bacterial suspensions were prepared in saline solution and used for subsequent infection experiments.

### Infection model

Infections were carried out as described previously [46]. Briefly, mice were anesthetized with intraperitoneal ketamine and xylazine injections. Three 0.5 cm scratches were made on the cornea with 25G needle tip and an inoculum of 1 x 10^7^ cfu of *P*. *aeruginosa* delivered in 5 μl onto the eye. Mice remained sedated for approximately 30 min. For evaluation of corneal pathology, daily scores were recorded by an observer unaware of the experimental status of the animals based on the following scoring system using a graded scale of 0 to 4 as follows: 0, eye macroscopically identical to the uninfected contra-lateral control eye; 1, faint opacity partially covering the pupil; 2, dense opacity covering the pupil; 3, dense opacity covering the entire anterior segment; and 4, perforation of the cornea, phthisis bulbi (shrinkage of the globe after inflammatory disease), or both. To determine corneal bacterial counts at 24h after infection, mice were sacrificed, the eyes were enucleated, and the corneas were dissected from the ocular surface. To quantify *P*. *aeruginosa* levels, corneas were suspended in PBS, 0.05% Triton X100, serially diluted and plated on *P*. *aeruginosa* selective McConkey agar plates.

### Reconstitution experiments

Mouse and human gut reconstituted GF mice were generated as previously described [47]. To generate monocolonized animals, 1000 CFU *CNS* sp. were placed onto the ocular surface of GF mice. Mice were rested for 2 weeks to allow for colonization prior to infection experiments. Ocular swabs were collected and plated on blood-agar plates and mannitol agar plates to determine the levels of ocular colonization.

### Eye washes

Ten μl PBS were instilled onto the ocular surface of each eye, pipetted up and down for five times and then pooled from both eyes. Protein levels from pooled eye washes of 4 to 6 individual mice were quantified using a standard Bradford reagent (BioRad), 20 μg of total protein were subjected to trypsin digestion and subsequent LC-MS/MS quantification (The Thermo Fisher Center for Multiplexed Proteomics) [48].

### Multiplexed quantitative mass spectrometry

Sample processing steps included SDS-PAGE purification of proteins, in-gel protein digestion using trypsin and peptide labeling with TMT 10-plex reagents. Multiplexed quantitative mass spectrometry data were collected on an Orbitrap Fusion mass spectrometer operating in a MS^3^ mode using synchronous precursor selection for MS^2^ fragment ion selection [49]. MS^2^ peptide sequence data were searched against a Uniprot mouse database with both the forward and reverse sequences using the SEQUEST algorithm. Further data processing steps included controlling peptide and protein level false discovery rates, assembling protein groups, and protein quantification from peptides. The p-values were calculated using Benjamini-Hochberg FDR correction [50–54].

### Histopathology examinations

Eyes were enucleated from euthanized mice, fixed in 4% (v/v) paraformaldehyde, and subsequently embedded in paraffin. Four μm sections were cut and stained with hematoxylin-eosin to visualize tissue morphology following previously used techniques [55]. The levels of ocular inflammation in the corneal sections was quantified on a scale of 1 to 4, with “1”being reflective of no neutrophil influx in the cornea or anterior chamber and healthy appearance; “2” denoting mild inflammation, preserved corneal epithelial layer, presence of neutrophils in the conjunctival tissues; “3” being reflective of moderate inflammation, loss of epithelial layer, influx of neutrophils in the corneal epithelium, less than 50 cells/ field of vision at 40X magnification, neutrophils lining the anterior chamber; and “4” denoting severe inflammation, lost corneal epithelial layer, massive influx of neutrophils in the cornea (more than 50 cells/field of vision at 40X magnification); numerous neutrophils present and scattered thought the anterior chamber. Histological scoring was carried out by Dr. Roderick Bronson, (HMS, Histopathology core) blindly using sections which did not display genotypic and phenotypic information.

### Cytokine analysis

Cytokine levels (IL-1ß, KC, IL-6) of corneal lysates were determined by commercially available ELISA assays (R&D Systems). In addition, IL-12p70, IL-10, IFN-γ were measured using a Meso Scale Discovery (MSD) multiplex 7-spot electrochemiluminescence (ECL) assay and outputs measured by an ultra-low noise charge-coupled device (CCD) Imager 2400 (Meso Scale Discovery, Gaithersburg, MD, USA). The MSD ECL platform has been previously validated against cytokine standards recommended by WHO and U.K. National Institute for Biological Standards and Control (NIBSC) and by comparison to traditional ELISA [56].

### Antibiotic treatments

Four week old SW SPF mice (Taconic) were treated with antibiotic cocktail in the drinking water containing carbenicillin (1g/L), neomycin (1g/L), metronidazole (1g/L), vancomycin (Henry Shein) (0.5g/L), and levaquin (0.15g/L) for 4 weeks [4,41,57]. One packet of a sucralose-based artificial sweetener (Splenda, Heartland Food Products Group) was used to make the antibiotics containing water palatable. Fecal pellets were collected before and every week during the antibiotic treatment to enumerate the intestinal cultivable aerobic and anaerobic bacteria. Antibiotic containing water was renewed every three days and animal cages were replaced every three days. Fecal samples were serially diluted and plated on blood, McConkey and mannitol agar plates in duplicates. Plates were incubated at 37°C both, aerobically and anaerobically.

To evaluate the impact of local microbiota on immunity to infection, separate cohorts of 8-week old, gender matched, SPF SW mice (Taconic) were treated with Gentak eye ointment (Patterson Veterinary) twice daily for four days. The control group was treated with sterile saline. Mice were rested for 4 days, and infections with *P*. *aeruginosa* were carried out as described above. Conjunctival swabs were collected before and after treatment to enumerate conjunctival bacterial presence.

### Purification of PMNs and bactericidal assays

Murine bone marrow was flushed from both hind limbs with PBS supplemented with 2% fetal bovine serum and 1 mM EDTA. The cells were washed, erythrocytes in the cell pellet were lyzed using the Mouse Erythrolysis Kit (R&D Systems) according to the manufacturer’s instruction, and neutrophils were isolated using the EasySep Mouse Neutrophil Enrichment Kit (Vancouver, Canada). Neutrophils were incubated with *P*. *aeruginosa* strain PA01 at an MOI of 100:1 for 90 min at 37°C on a rotator. Aliquots taken at time 0 and 90 min were serially diluted and plated on McConkey agar to determine numbers of live *P*. *aeruginosa*. Percentage of killing ability of neutrophils was calculated as in [58].

### Isolation of RNA, quantitative PCR analysis, and RNA seq analysis

RNA was isolated from 5 x 10^6^ neutrophils using TRIzol-chloroform (Invitrogen) precipitation as per the manufacturer’s protocol. The RNA precipitate was cleaned using an RNeasy mini kit (Qiagen) that included a DNA clean-up step. Total RNA concentration and integrity was assessed using the Agilent 2100 Bioanalyser RNA Nano chip. Samples that had RNA integrity (RIN) ≥9.0 were used for transcriptomic analysis. The Biopolymer Facility, Harvard Medical School, performed RNA sequencing on Illumina HiSeq2500 rapid mode with V2 reagents in 50bp single-end format. The reads were mapped to the recent mouse genome reference (GRCm38.p4) on CLC Genomics Workbench Version 7.5.1. Transcripts were assembled and normalized using RNA-Seq module and Expression analysis module in CLC Genomics Workbench. Statistical analysis was performed on normalized expression values from the replicates of each group using the EdgeR Bioconductor module [59]. The upstream regulators of the differentially expressed genes were analyzed through the use of QIAGEN’s Ingenuity Pathway Analysis (IPA, QIAGEN Redwood City, www.qiagen.com/ingenuity).

In additional series of experiments, total RNA from conjunctival samples were extracted using TRIzol (Ambion, LifeTechnologies) method. Samples were treated with DNase I (Qiagen, Germany) and cleaned using RNAeasy (Qiagen, Germany) mini columns. Quantitative PCR was performed on 250 ng of total RNA with a one-step reverse transcription (RT)-PCR kit, Power SYBR Green RNA-to-CT1-Step (Applied Biosystems) on CFX Connect Real-Time PCR Detection System (BioRad). Expression levels of the target genes were normalized to GAPDH. Relative fold differences were calculated by -2^ΔΔC^_T_ method [60]. The following primer set was used to measure IL-1ß expressions: IL-1ß, F: 5′-CCATGGCACATTCTGTTCAAA-3′, R: 5′-GC-CCATCAGAGGCAAGGA-3′ [61].

### Statistical analysis

Statistical analysis of corneal pathology scores, bacterial burden, and cytokine levels were either by Mann-Whitney U test for pair-wise comparisons or the Kruskal-Wallis non-parametric ANOVA with Dunn’s correction for Multigroup comparisons and individual 2-group comparisons (Prism 4.0 for Macintosh). For the purposes of the analysis of the LC-MS^3^ data and bactericidal activity assays the Unpaired Student’s *t*-test was used. Differences were considered significant if the p value was <0.05 (Prism 4.0 for Macintosh).

## Supporting Information

S1 Fig Hematoxylin and eosin staining of eye balls derived from non-infected SW GF and SPF mice. The eye tissues were fixed in formalin, excised from the animals, paraffin embedded, sectioned, and stained with hematoxylin and eosin to visualize morphology. No gross morphological differences were observed when slides were viewed at 40x.
(EPS)

S2 Fig Relative protein levels measured in eye washes from GF, SPF, *P*. *aeriginosa* 6294-infected GF and SPF mice. **A.** The total protein levels were measured in eye washes harvested from GF (n = 15), SPF (n = 15), infected with *P*. *aeruginosa* 6294 GF (n = 10) and SPF (n = 10) mice via Bradford. p-values were generated using one-way ANOVA. There were no significant differences in the total protein levels measured in non-infected GF and SPF SW samples. Infected GF-derived ocular washes showed elevated total protein presence when compared to baseline. While there was a tendency for the infected SW mice to have lower total protein levels in the eye washes, this difference did not reach significance. **B.** Five micrograms total protein from GF, SPF, *P*. *aeruginosa* 6294-infected GF, and *P*. *aeruginosa* 6294-infected SPF eye washes were used for tryptic in-gel digest. The resultant material was visualized by Coomassie Brilliant Blue staining. Each sample is presented by biological duplicates. The image shows that comparable levels of total protein were used for the LC-MS^3^ analysis.
(EPS)

S3 Fig PMNs derived from GF mice have significantly decreased bactericidal activities against *P*. *aeruginosa PA14*. Percent of *P*. *aeruginosa* PA14 killing by GF and SPF SW-derived PMNs *in vitro* (p = 0.02, unpaired Student *t*-test).
(EPS)

S4 Fig GF-derived PMNs release less reactive oxygen species (ROS) in response to *P*. *aeruginosa* 6294. 0.5 to 1 x 10 ^6^ purified murine PMNs per sample were exposed to *P*. *aeruginosa* in the presence of horse radish peroxidase (Sigma) and luminol. Released ROS were monitored for up to 1h using a TECAN luminescence reader [62]. p-value by One-way ANOVA.
(EPS)

S1 Table List of all identified proteins from the ocular washes in GF and SPF mice. (XLSX)

S2 Table Significantly decreased proteins in the ocular washes of GF mice when compared to SPF SW mice identified by the LC-MS/MS analysis. UniProt protein IDs, gene symbols, description, spectral counts, fold change, and Benjamini-Hochberg FDR corrected p-values are listed. The listed proteins were identified with more than 4 unique peptides cut-off and showed more than 1.75-fold change in the expression levels.
(XLSX)

S3 Table Significantly increased proteins in the ocular washes of GF and SPF mice identified by the LC-MS/MS analysis. UniProt protein IDs, gene symbols, description, spectral counts, fold change, and Benjamini-Hochberg FDR corrected p-values are listed. The listed proteins were identified with more than 4 unique peptides cut-off and showed more than 1.75-fold change in the expression levels.
(XLSX)

S4 Table Significantly increased proteins in the ocular washes of infected GF and SPF mice identified by the LC-MS/MS analysis. UniProt protein IDs, gene symbols, description, spectral counts, fold change, and Benjamini-Hochberg FDR corrected p-values are listed. The listed proteins were identified with more than 4 unique peptides cut-off and showed more than 1.75-fold change in the expression levels.
(XLSX)

S5 Table List of transcripts differentially present in SPF versus GF-derived neutrophils. (XLSX)
